# In Vitro Analysis of Human Cartilage Infiltrated by Hydrogels and Hydrogel-Encapsulated Chondrocytes

**DOI:** 10.3390/bioengineering10070767

**Published:** 2023-06-26

**Authors:** Hannah Köck, Birgit Striegl, Annalena Kraus, Magdalena Zborilova, Silke Christiansen, Nicole Schäfer, Susanne Grässel, Helga Hornberger

**Affiliations:** 1 Biomaterials Laboratory, Faculty of Mechanical Engineering, Ostbayerische Technische Hochschule (OTH), 93053 Regensburg, Germany; hannah.koeck@klinik.uni-regensburg.de; 2 Department of Orthopaedic Surgery, Experimental Orthopaedics, Centre for Medical Biotechnology (ZMB/Biopark 1), University of Regensburg, 93053 Regensburg, Germany; nicole.schaefer@klinik.uni-regensburg.de (N.S.); susanne.graessel@klinik.uni-regensburg.de (S.G.); 3 Regensburg Center of Biomedical Engineering (RCBE), Ostbayerische Technische Hochschule (OTH) and University of Regensburg, 93053 Regensburg, Germany; birgit.striegl@oth-regensburg.de; 4 Institute for Nanotechnology and Correlative Microscopy eV INAM, 91301 Forchheim, Germany; annalena.kraus@inam-forchheim.de (A.K.); schrist1@gwdg.de (S.C.); 5 Department of Orthopaedic Surgery, University of Regensburg, 93053 Regensburg, Germany; m.zborilova@asklepios.com

**Keywords:** osteoarthritis, human articular cartilage, chondrocytes, infiltration, zwitterionic monomers, hydrogels

## Abstract

Osteoarthritis (OA) is a degenerative joint disease causing loss of articular cartilage and structural damage in all joint tissues. Given the limited regenerative capacity of articular cartilage, methods to support the native structural properties of articular cartilage are highly anticipated. The aim of this study was to infiltrate zwitterionic monomer solutions into human OA-cartilage explants to replace lost proteoglycans. The study included polymerization and deposition of methacryloyloxyethyl-phosphorylcholine- and a novel sulfobetaine-methacrylate-based monomer solution within ex vivo human OA-cartilage explants and the encapsulation of isolated chondrocytes within hydrogels and the corresponding effects on chondrocyte viability. The results demonstrated that zwitterionic cartilage–hydrogel networks are formed by infiltration. In general, cytotoxic effects of the monomer solutions were observed, as was a time-dependent infiltration behavior into the tissue accompanied by increasing cell death and penetration depth. The successful deposition of zwitterionic hydrogels within OA cartilage identifies the infiltration method as a potential future therapeutic option for the repair/replacement of OA-cartilage extracellular suprastructure. Due to the toxic effects of the monomer solutions, the focus should be on sealing the OA-cartilage surface, instead of complete infiltration. An alternative treatment option for focal cartilage defects could be the usage of monomer solutions, especially the novel generated sulfobetaine-methacrylate-based monomer solution, as bionic for cell-based 3D bioprintable hydrogels.

## 1. Introduction

Human articular cartilage (AC) is a unique tissue composed of extracellular matrix (ECM), which mainly consists of water, type II collagen, negatively charged proteoglycans, and chondrocytes embedded within it. AC tissue can be divided vertically into superficial (SZ), transitional (TZ) and deep zones (DZ) and exhibits zonal mechanical as well as structural differences depending on the prevalent type and fibrillar arrangement of collagens, type of proteoglycans and water content. Articular cartilage has a very limited intrinsic regenerative capacity partly due to the lack of vascularization and innervation [1,2].

Thus, it remains challenging to identify successful long-term treatment options for AC defects. Osteoarthritis (OA) is one of the most prevalent age-related and/or trauma-induced musculoskeletal diseases of the articular joint, characterized by cartilage degradation, synovial inflammation, subchondral bone sclerosis, ligament calcification and osteophyte formation [3]. The main risk factors besides age and joint injuries following traumata are obesity, gender and genetics [4]. OA can lead to the irreversible destruction of AC and other joint tissues, resulting in pain, swelling, inflammation and gradual stiffness of the joint [5,6]. Due to the increasing prevalence of OA [7] and the significant limitations on the quality of life of affected patients, novel therapeutic approaches such as disease-modifying OA drugs (DMOADs) or regenerative therapies are urgently sought to achieve long-lasting symptomatic relief [8].

Current treatment options mainly focusing on pain improvement are non-steroidal anti-inflammatory drugs (NSAIDs), opioids, corticosteroids and other DMOADs [8,9,10,11]. Total joint replacement with an artificial prosthesis is still considered as the gold standard in terms of recovering joint function, pain relief and quality of life, especially in patients with progressive OA [8,12]. Instead of total or partial joint replacement, successful cartilage structure-preserving treatments would be preferable. Currently, focal cartilage lesions are surgically treated by microfracturing of the subchondral bone (bone-marrow stimulus), chondroplasty (removal of damaged tissue) and transplantation of cartilage autografts or allografts [13]. Other promising treatment options for trauma-induced cartilage defects are cell-based therapies, such as autologous chondrocyte implantation (ACI); mesenchymal stem cells (MSCs) and induced pluripotent stem cells (iPSCs) are potential candidates for application in the future treatment of cartilage lesions. The number of autologous obtainable primary chondrocytes is limited; thus, stem cell therapies (MSCs or iPSCs) are of particular interest due to their pluripotent properties, allowing chondrogenic differentiation. However, the use of allogenic MSCs in clinical applications remains challenging due to several obstacles, including control of MSC differentiation and maintenance of growth and passaging in vitro, immunogenicity and cell isolation from human tissues. Some studies show great in vivo and in vitro potential of MSCs to generate hyaline cartilage, though other studies suggest the adoption of a hypertrophic phenotype that precedes endochondral ossification, a process that is not found in healthy articular cartilage and thus prevents the formation of a stable chondrogenic phenotype [9,14,15,16,17].

To date, therapies that ensure the long-term functionality of macromolecular cartilage matrix components are lacking, as the characteristic supramolecular structure of the cartilage tissue cannot yet be replicated and thus the biomechanical stability of the tissue is not restored [18]. This problem has led to the development of approaches based on hydrogel scaffolds that combine materials, cells and bioactive factors to create new opportunities for biomechanically functioning tissue replacement [19]. Hydrogels consist of unique three-dimensional (3D) polymeric substances which are crosslinked at interconnection points [20,21]. Due to their mesh structure and water-binding capacity, hydrogels are particularly interesting for cartilage repair/replacement or as a supportive biomaterial [21,22]. They enable material exchange and encapsulation of cells [23] (e.g., chondrocytes, MSCs [24]), bio-macromolecules (e.g., peptides, proteins, saccharides [25]), active ingredients (e.g., loxoprofen [26], curcumin [27], rapamycin [28]) and drug delivery systems [29] (Xianqin Tong et al., 2019). Natural biomaterials (e.g., collagens, hyaluronic acid) offer superior potential for cell adhesion and biocompatibility, whereas synthetic biomaterials (polymers, peptides) excel mainly in terms of definable biomechanical requirements [30], although naturally based flexible hydrogels with adjustable mechanical properties have already been developed [31] (Qi et al., 2020). In this context, biofouling, i.e., microbial contamination of biomaterial surfaces, has an important impact, as microbial contamination can lead to inflammatory responses in the recipient tissue [32,33]. In general, non-fouling materials usually exhibit hydrophilic behavior [34]. Therefore, zwitterionic-based hydrogels have attracted attention in research over recent years, as they exhibit outstanding anti-fouling properties [33,35,36]. Zwitterionic polymers are characterized by their equal numbers of anionic and cationic groups. The most common of these hydrogels are based on polysulfo-, polycarboxy- or polyphosphobetaine. When zwitterionic hydrogel surfaces are destroyed, the zwitterionic pairs attract electrostatically, resulting in a self-healing capacity [36].

In addition, zwitterionic polymers exhibit excellent lubricating properties [37], which is important in the context of articular cartilage function and pain-free movement. The loss of the unique lubricity of healthy cartilage surfaces and the associated low friction between joint surfaces is an important factor in the pathogenesis of OA. Increased friction results in increased wear and pro-catabolic cell effects that promote OA progression [38].

Several studies have already achieved significant reductions in friction and the restoration of cartilage-like lubricant properties by using zwitterionic polymers [39,40]. From a biomechanical point of view, AC is composed of a complex and zone-dependent ECM composite, in which collagen fibrils are responsible for the tensile strength and hydrated proteoglycans for elasticity and compressive strength, allowing high load-bearing capacity and low friction as a prerequisite for smooth movements [41,42]. The hydrophilic glycosaminoglycan (GAG) chains of the proteoglycans are responsible for the compression and recovery properties of the cartilage dimensions, as bound water molecules are forced out upon compression and osmotically attracted upon release [43]. Loss of GAGs is an early marker of OA and the concomitant loss of mechanical stability [44].

Several studies identified correlations between reduced GAG content and decreased lubricity [45]. In an approach to recover the lost GAG content in the early stages of OA, infiltration and polymerization of a 2-methacryloyloxyethyl phosphorylcholine (MPC)-based zwitterionic hydrogel in bovine OA-structures was found to improve lubrication and to increase mechanical stability [46,47]. This infiltration procedure as a natural–synthetic GAG-replacement hybrid system suggests a potential treatment option for early OA [47]. However, the response of human articular cartilage to an MPC-based hydrogel treatment has not yet been investigated.

Based on the promising results in terms of anti-fouling, lubricating and mechanical properties of zwitterionic polymers as cartilage ECM support materials, the aim of this in vitro study was to evaluate the in situ infiltration behavior of MPC- and novel sulfobetaine methacrylate (SBMA)-based hydrogels and to explore their effect on cell viability of infiltrated human OA-cartilage explants and on isolated human OA chondrocytes. In comparison to many other studies, the focus here was on human, naturally degraded osteoarthritic articular cartilage explants, designed to preserve the patient’s native cartilage and mimic the degenerated structures through in situ infiltration and polymerization, rather than to replace the endogenous cartilage.

## 2. Materials and Methods

### 2.1. Isolation and Cultivation of Human OA-Cartilage Explants and Isolated OA Chondrocytes

Cartilage explants: Experiments for this study were performed by using human cartilage explants and isolated chondrocytes from knee joints of OA patients (Table S1 in Supplementary Materials) that had been removed during knee replacement surgery. The use of human tissue was approved by the ethics committee at the University of Regensburg (ethics vote: 25-101-0189). For cartilage explant tissue culture, OA-cartilage plugs were extracted from the subchondral bone by using a scalpel and a biopsy punch with an 8 mm diameter. Subsequently, each plug was transferred to a sterile 24-well plate and immediately supplied with 2 mL Dulbecco´s Eagle´s medium/F12 (DMEM/F12), supplemented with 10 wt% fetal calf serum (FCS) and 1 wt% penicillin–streptomycin (Sigma-Aldrich, Taufkirchen, Germany). Cartilage explant plugs were cultured at 37 °C, 5 *v*/*v*% CO_2_ and 95% humidity until further use.

Chondrocytes: Chondrocytes were isolated as published previously [48]. Cartilage from OA patients was removed from the subchondral bone and cut into small pieces. Cartilage was digested with sterile filtrated 0.2% type II collagenase (Worthington, Lakewood, CA, USA) in DMEM/F12 supplemented with 1 wt% penicillin–streptomycin by shaking the solution for 16 h at 37 °C. Isolated chondrocytes were seeded at a density of 6000–12,000 cells/cm^2^ and expanded in DMEM/F12 medium supplemented with 10 wt% FCS and 1 wt% penicillin–streptomycin. Isolated chondrocytes were cultured at 37 °C, 5 *v*/*v*% CO_2_ and 95% humidity until further use. For all further experiments, chondrocytes at passage 1 were used.

### 2.2. Synthesis of MPC and SBMA Hydrogels

Three different monomer mixtures were prepared and are denoted as follows: MPC60, MPC30 and SBMA60. The MPC60 monomer solution was prepared according to the method described by Cooper et al. [47]. The monomer solution consists of 400 mOsm saline solution (Carl Roth, Karlsruhe, Germany) containing 0.6 g/mL MPC monomer, the crosslinker 1% mol/mol ethylene glycol dimethacrylate (EGDMA), and a photoinitiating system consisting of 115 mM triethanolamine (TEOA), 94 mM N-vinylpyrrolidone (NVP) and 0.1 mM Eosin Y (Merck Millipore, Darmstadt, Germany).

The synthesis of MPC30 and SBMA60 monomer solutions was performed in the same way, except the monomer component 0.6 g/mL MPC was replaced by 0.3 g/mL MPC (MPC30) and 0.6 g/mL SBMA (SBMA60) (Merck Millipore, Darmstadt, Germany), respectively. The monomer solutions were stored at 5 °C and protected from light exposure to avoid premature polymerization. The monomer solutions were polymerized into hydrogels for 10 min using a Bluephase G4 polymerization lamp (Ivoclar vivadent, Ellwangen, Germany) at 515 nm and an intensity of 1.200 mW/cm^2^, which guaranteed precise intensity. A Translux Energy lamp (Heraeus Kulzer GmbH & Co. KG, Hanau, Germany) at 515 nm and 900 mW/cm^2^ was applied only for the fluorescence study.

### 2.3. Preparation of Cartilage–Hydrogel and Chondrocyte–Hydrogel Composites

Cartilage–hydrogel composite: Human OA-cartilage tissue plugs were incubated in and infiltrated with monomer solutions (MPC30, MPC60 or SBMA60) at 37 °C, 5 *v*/*v*% CO_2_ and 95% humidity protected from light for 24 h. In addition, infiltration behavior with shortened infiltration times of 1, 5, 10 and 30 min in OA-cartilage explants was investigated exemplarily for the MPC60 monomer solution. After infiltration, the cartilage–hydrogel composites were irradiated with visible light (VL) for 10 min. After polymerization of monomers, all samples were rinsed three times with phosphate-buffered saline (PBS) (Thermo Fischer, Kandel, Germany) and cultured in DMEM/F12 medium supplemented with 10 wt% FCS and 1 wt% penicillin–streptomycin for 1 and 3 days.

For single-component and parameter analysis, OA-cartilage explants were infiltrated with individual components of the monomer solutions for 24 h. For this, components of the monomer solutions were dissolved separately in saline solution at the appropriate concentrations. Furthermore, untreated OA-cartilage explants (controls) were irradiated with VL for 10 min.

Chondrocyte–hydrogel composite: Isolated human OA chondrocytes (500,000 cells) were embedded in 50 µL of the respective monomer solution (MPC30, MPC60 or SBMA60). The cell–hydrogel construct was formed by crosslinking with VL for 10 min. The polymerized chondrocyte-containing scaffolds were incubated in DMEM/F12 medium supplemented with 10 wt% FCS and 1 wt% penicillin–streptomycin at 37 °C, 5 *v*/*v*% CO_2_ and 95% humidity for 1, 3 and 7 days.

### 2.4. Fourier-Transform Infrared Spectroscopy (FTIR)

Monomers (MPC and SBMA), hydrogels (MPC30, MPC60 and SBMA60), human OA-cartilage samples (control, without hydrogel) and OA-cartilage–hydrogel (MPC30, MPC60 and SBMA60) constructs were analyzed with FTIR to verify successful polymerization and infiltration. The outermost layer of the infiltrated OA-cartilage plugs was removed to exclude purely superficial deposition of the infiltrating hydrogels. All OA-cartilage samples were fixed with 4 wt% PFA, subjected to an ethanol gradient (50 wt%, 70 wt%, 96 wt% and 100 wt%) and washed with PBS. Hydrogel cylinders, infiltrated OA cartilage and non-infiltrated cartilage plugs (control) were dried to a constant weight. The potassium bromide (Merck Millipore, Darmstadt, Germany) pellet method was used to identify functional groups using an FTIR spectrometer (Tensor 27, Bruker, MA, USA). The evaluation was performed with Opus Viewer (Bruker Corporation, Billerica, MA, USA).

### 2.5. Scanning Electron Microscopy (SEM)

The microstructure of human OA cartilage was studied with SEM and compared with cartilage–hydrogel composite samples, with MPC60-containing explants serving as examples. Samples were prepared with some modifications following the Electron Microscope Unit procedure [49] and the Biological Sample Fixation for SEM protocol [50], as described below. OA-cartilage samples were infiltrated with MPC60 monomer solution and polymerized as described above. After infiltration and polymerization, they were cultured in DMEM/F12 medium supplemented with 10 wt% FCS and 1 wt% penicillin–streptomycin for 24 h at 37 °C. For SEM imaging, prepared human OA cartilage samples containing MPC60 hydrogel and control human OA-cartilage samples were fixed by immersion in 2 wt% glutaraldehyde solution (Merck Millipore, Darmstadt, Germany) dissolved in 0.1 M sodium cacodylate buffer (pH 7.4) (Alfa Aesar, Ward Hill, MA, USA) for 24 h at room temperature. The OA-cartilage samples were then washed two times with 0.1 M sodium cacodylate puffer, 15 min per wash. They were post-fixed in 1 wt% osmium tetroxide (Merck Millipore, Darmstadt, Germany) dissolved in sodium cacodylate buffer (pH 7.4) for 2 h. After post-fixation, the samples were washed three times with deionized water, 5 min per wash. They were dehydrated for 5 min with a specific series of ethanol (Carl Roth, Karlsruhe, Germany) (50 wt%, 70 wt% and 95 wt%) and two times for 10 min with 100 wt% ethanol. The samples were dried using a chemical drying method with 1, 1, 1, 3, 3, 3-Hexamethyldisilazan (HMDS) (Acros Organics/Thermo Fisher Scientific, Waltham, MA, USA). Dried OA-cartilage samples were immersed in a mixture of ethanol and HMDS (2:1, 1:1, 1:2) for 15 min in respective concentrations followed by three immersions for 15 min each in pure HMDS; the last samples in HMDS were then left under a fume hood for 24 h. After drying, the cartilage samples were attached to aluminum stubs and viewed using a scanning electron microscope (1.00 kV, Auriga 40, Carl Zeiss Microscopy GmbH, Jena, Germany).

### 2.6. Fluorescence Microscopy

In order to investigate the infiltration behavior of the MPC and SBMA monomer solutions in OA-cartilage explants, the fluorescent monomer methacryloyloxyethyl thiocarbamoyl rhodamine B (MTR) (Polysciences Inc., Warrington, PA, USA) was added to the monomer solutions MPC30, MPC60 or SBMA60 (1:100). Following incubation, infiltration and polymerization, the hydrogel–cartilage plugs were fixed with 4 wt% paraformaldehyde (PFA) (Sigma-Aldrich, Taufkirchen, Germany) for 24 h. After fixation, the samples were washed three times in PBS, 5 min each, embedded in TissueTek (Sakura Finetek Germany GmbH, Umkirch, Germany) and frozen for at least 24 h at −80 °C. Thereafter, embedded samples were cut into 10 µm thick cryosections using a microtome (CM 1950, Leica Camera, Wetzlar, Germany). After rehydration in PBS for 5 min, cell nuclei were counterstained with DAPI (1:1000 in PBS, 10 min, protected from light). Subsequent to the final washing steps, sections were covered with Dako Fluorescence Mounting Medium (Agilent Technologies Inc., Santa Clara, CA, USA) and dried for several hours at 4 °C. Images were taken with an all-in-one fluorescence microscope (Keyence BZ-X810, Nikon, Tokyo, Japan).

### 2.7. Live/Dead Cell Staining of Encapsulated Chondrocytes in Hydrogels and Cartilage–Hydrogel/Component Composites

Chondrocyte–hydrogel composite: Isolated human OA chondrocytes were encapsulated for 1, 3 or 7 days in different zwitterionic hydrogels (MPC60 and SBMA60 hydrogels) to determine potential cytotoxic effects by using a LIVE/DEAD Viability/Cytotoxicity kit (Invitrogen/Thermo Fisher Scientific, Waltham, MA, USA). Living cells were stained with calcein-AM (Ca-AM), and dead cells were stained with ethidium homodimer-1 (EthD-1). The samples were washed with PBS and afterward incubated in a staining solution containing 2 µM Ca-AM and 2 µM EthD-1 in PBS in a 6-well plate for 30 min protected from light at 37 °C, 5 *v*/*v*% CO_2_ and 95% humidity. Subsequently, the hydrogel–chondrocyte scaffolds were washed three times with PBS, applied onto slices, covered with cover glass and imaged with a confocal laser scanning microscope (Eclipse E600, Nikon, Japan). Z-stack images were taken using EZ-C1 software (Nikon, version 3.91) at 10× and 20× magnification (z step size = 0.5 µm, approx. 80–100 slices per sample, approx. 50–60 µm deep). The evaluation was performed with ImageJ/Fiji (National Institutes of Health, Bethesda, MD, USA).

Cartilage–hydrogel/component composite: Potential cytotoxic effects of infiltrated (24 h) and polymerized monomer solutions (MPC30, MPC60, SBMA60) in human OA-cartilage explants and the individual components of the monomer solutions (24 h infiltration) were analyzed by using a LIVE/DEAD Viability/Cytotoxicity kit. MPC60-containing cartilage samples with reduced infiltration times (1, 5, 10 and 30 min) were also investigated by using the LIVE/DEAD Viability/Cytotoxicity kit. Cross-sectional slices (<0.5 mm) of the hydrogel–cartilage plugs were prepared using a blade. Slices were applied to a cannula and covered with 1.5 mL LIVE/DEAD staining solution containing 2 µM Ca-AM and 2 µM EthD-1 in PBS within a 15 mL falcon for 24 h protected from light at 37 °C, 5 *v*/*v*% CO_2_ and 95% humidity. After incubation, cartilage slices were washed three times with PBS and imaged with a confocal laser scanning microscope (Eclipse E600, Nikon, Japan). Z-stack images were taken at 4×, 10× and/or 20× magnification (z step size = 0.5 µm, approx. 80–100 slices per sample, approx. 50–60 µm deep). The evaluation was performed by using ImageJ/Fiji.

### 2.8. CellTiter-Blue (CTB) Viability Assay

CellTiter-Blue Cell Viability Assay (Promega, Madison, WI, USA) was used to evaluate the metabolic activity of hydrogel-containing human OA-cartilage plugs. Human cartilage plugs were covered with 30 µL CellTiter-Blue reagent and 300 µL DMEM/F12 supplemented with 10 wt% FCS and 1 wt% penicillin–streptomycin for 5 h at 37 °C, 5 *v*/*v*% CO_2_ and 95% humidity. For the measurement, 100 μL protrusion of the used working solution was placed in a black 96-well plate to be analyzed with a Tecan ELISA reader (GENios, Maennedorf, Switzerland) with excitation of 560 nm and emission of 590 nm.

### 2.9. Statistical Analysis

Statistical analysis was performed using Prism8.0.2 software (GraphPad Software, San Diego, CA, USA). Nonparametric, paired *t*-test (Mann–Whitney) or Kruskal–Wallis test (Dunn´s multiple comparison) was used to compare the results. All data are expressed as the mean ± standard deviation (SD).

## 3. Results

The ex vivo infiltration of zwitterionic monomer solutions into OA-cartilage plugs and their in situ polymerization were investigated. Three zwitterionic monomer solutions (MPC30, MPC60 and SBMA60) and their hydrogel synthesis and infiltration behavior in OA-cartilage explants were investigated in detail.

### 3.1. Successful Polymerization and Infiltration of Monomer Solutions into Human OA-Cartilage Plugs

**Figure 1:**
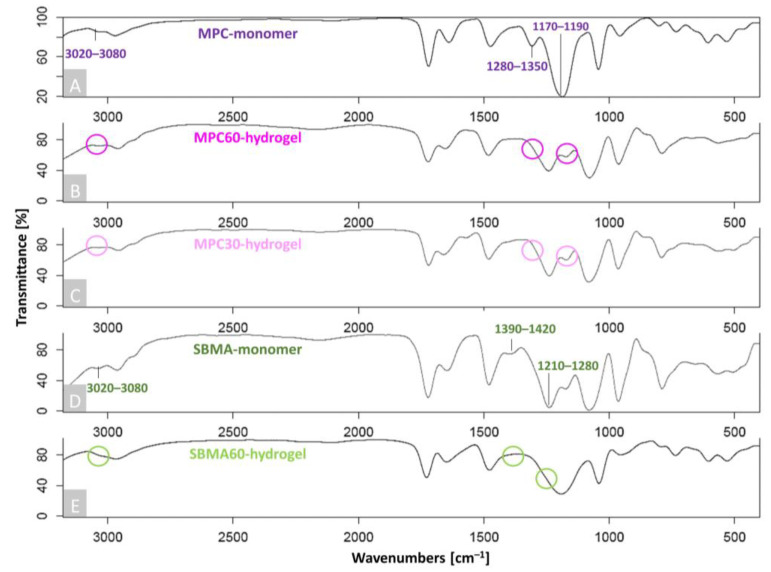
FTIR spectra of MPC60-, MPC30- and SBMA60-hydrogel synthesis. Shown are characteristic absorption bands of (**A**) MPC monomer, (**B**) MPC60 hydrogel, (**C**) MPC30 hydrogel, (**D**) SBMA monomer and (**E**) SBMA60 hydrogel. Peaks which indicated the monomer bands have disappeared or shifted towards the polymerized hydrogel bands, confirming successful hydrogel polymerization, which is indicated by squiggles; n = 5.

Hydrogel synthesis of the monomer solutions (MPC30, MPC60 and SBMA60) was revealed as early as after 10 min irradiation with VL (515 nm) by a characteristic color change from red to yellow, as the reddish Eosin Y was activated. The polymerization of the MPC60 and SBMA60 monomer solutions resulted in a stable and high-viscosity hydrogel, whereas the MPC30 monomer solutions formed a low-viscosity hydrogel. FTIR was performed in addition to the optical analysis to confirm the polymerization of MPC and SBMA monomer solutions to MPC60, MPC30 and SBMA60 hydrogels. For this, the FTIR spectra of the pure MPC and SBMA monomer powders (Figure 1A,D) and those of the synthesized MPC60-, MPC30- and SBMA60-hydrogel samples (Figure 1B,C,E) were compared. The highly reduced occurrence of the specific peak between 3020 and 3080 cm^–1^ and the shifted bands at 1280–1350 cm^–1^ and 1170–1190 cm^–1^ in the MPC-containing hydrogels (Figure 1B,C) confirmed the successful polymerization of MPC30 and MPC60 hydrogels from the MPC monomers (Figure 1A). The absence of the absorption bands at 3020–3080 cm^–1^ and 1390–1420 cm^–1^ and the displaced vibrations between 1210–1280 cm^–1^ in the spectra of the SBMA hydrogel (Figure 1E) compared to the FTIR spectra of the SBMA monomer (Figure 1D) indicated successful polymerization of SBMA60 hydrogels.

**Figure 2:**
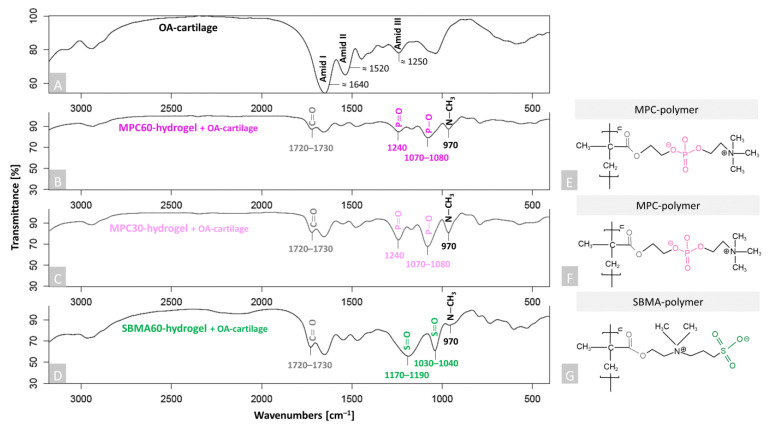
FTIR analysis of non-infiltrated and infiltrated human articular OA-cartilage plugs. (**A**) Human non-infiltrated OA-cartilage plug (control) (black). In the spectrum, the Amides I–III, which are characteristic of collagen fibrils within the cartilage tissue, are marked accordingly. (**B**–**D**) Polymerized human OA-cartilage plugs infiltrated with MPC60 (**B**, pink), MPC30 (**C**, light pink) or SBMA60 (**D**, green) monomer solution. (**E**–**G**) Characteristic functional groups and chemical structures are highlighted as evidence of successful infiltration: (**E**,**F**) zwitterionic MPC polymers with P–O and P=O for phosphate group of MPC (pink); (**G**) SBMA polymer with S=O for sulfonate group of SBMA (green). C=O for ester group (**B**–**G**, gray) and N–CH_3_ for choline group (**B**–**D**, black). n = 6. Created with KingDraw.com.

To study the deposition of all three hydrogels (MPC30, MPC60 and SBMA60) into the human OA-cartilage ECM, FTIR was used (Figure 2) and specific spectra for cartilage samples were detected. The characteristic peaks of non-infiltrated OA cartilage (control) were assigned to Amid I (≈1640 cm^–1^), Amid II (≈1520 cm^–1^) and Amid III (≈1250 cm^–1^), which are significant for collagen fibrils of cartilage tissue [51,52,53] (Figure 2A). Amid I–III peaks also appear in the spectra of the infiltrated OA-cartilage samples, but these are not explicitly marked again (Figure 2B,C). Two significant peaks around 1720–1730 cm^–1^ and 970 cm^–1^ were detected in the spectra of the infiltrated OA-cartilage samples containing MPC60, MPC30 and SBMA60 hydrogels (Figure 2B–D), compared to the non-infiltrated OA-cartilage sample (Figure 2A). The vibrations at 1720–1730 cm^–1^ can be ascribed to C=O stretching (ester group of hydrogels, Figure 2B–G, grey), and the peaks around 970 cm^–1^ are characteristic of N-CH_3_ (choline group of MPC hydrogels and the choline-type group of SBMA hydrogels, Figure 2B–G, black). These peaks are found in the respective five investigated hydrogel-containing OA-cartilage samples, as these functional groups are related to the identical chemical compounds of the (Figure 2E–G). Depending on the polymer component (MPC or SBMA), it was possible to identify additional specific peaks. Cartilage samples containing MPC hydrogels exhibited a characteristic peak at 1240 cm^–1^, which belongs to P=O stretching, and oscillations around 1070–1080 cm^–1^, which are assigned to P–O stretching of the phosphate functional group of MPC (MPC60: Figure 2B,E pink and MPC30: Figure 2C,F light pink) [54]. In cartilage samples containing SBMA60 hydrogel, specific peaks are located between 1030 and 1040 cm^–1^ and between 1170 and 1190 cm^–1^, which belong to S=O symmetric stretching and S=O asymmetric stretching vibrations of the sulfonate groups of SBMA (Figure 2D,G green) [51,55]. However, it should be noted that the peak of Amid III (1250 cm^–1^) correlates with P=O (1240 cm^–1^), but the Amid III vibration is superimposed by the characteristic vibrations of the polymers. These data confirm the successful infiltration and deposition of all tested hydrogels throughout the human OA-cartilage explants.

**Figure 3:**
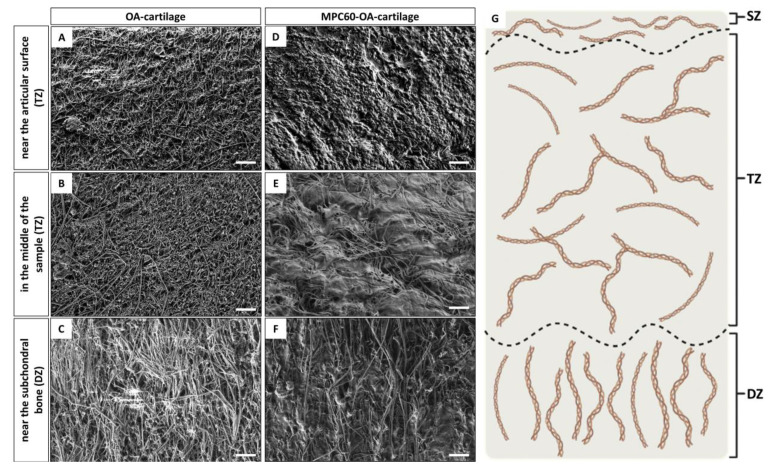
SEM cross-sections of human OA cartilage with and without hydrogel. Collagen fibers of non-infiltrated OA-cartilage ECM (**A**) near the articular surface, (**B**) in the middle of the cartilage sample and (**C**) near the subchondral bone. Collagen fibers of MPC60-containing OA-cartilage ECM (**D**) near the articular surface, (**E**) in the middle of the cartilage sample and (**F**) near the subchondral bone. The assignment of the images is based on the orientation of the collagen fibrils: (**A**,**D**) the randomly orientated collagen fibers near the articular surface can be assigned to the transitional zone (TZ), (**B**,**E**) the randomly orientated collagen fibers in the middle OA-cartilage sample can be assigned to the transitional zone (TZ) and (**C**,**F**) the collagen fibers orientated perpendicular to the articular surface can be assigned to the deep zone (DZ). (**D**–**F**) Successful infiltration from hydrogel deposits within human OA-cartilage ECM structures is demonstrated in all regions. n = 6; magnification 5000×. Scale bar 2 µm. (**G**) A schematic representation of articular cartilage collagen fibril organization with the specific classification into SZ, TZ and DZ according to the alignment of the collagen fibers. Created with BioRender.com.

Initially, SEM was used to study ex vivo OA-cartilage structures without hydrogel infiltration (Figure 3A–C). Thereby, the cartilage tissue cross-sections have been systematically investigated in three regions: near the articular surface (superficial zone; Figure 3A), in the middle of the cross-section (middle/transitional zone; Figure 3B) and near the subchondral bone (deep zone; Figure 3C). In each zone of the OA-cartilage explant, fibrillar structures and their orientation could be identified. The fibers are mostly randomly orientated; however, in the deep zone near the subchondral bone, the fibers are orientated perpendicular to the articular surface. All fibers represent collagen fibrils, and the orientation of collagen fibers determines typically the zone category of articular cartilage tissue, consisting of the superficial zone (SZ) middle/transitional zone (TZ) and deep zone (DZ) [56,57]. The SZ adjacent to the articular surface is characterized by an arrangement of the collagen fibers parallel to the surface, the TZ in the middle of the cartilage tissue is characterized by randomly orientated fibers and the DZ adjacent to the subchondral bone is characterized by collagen fibers orientated perpendicular to the articular surface (Figure 3G). SEM revealed TZ and DZ structures; however, SZ structures could not be identified in most of the analyzed OA-cartilage explants, and the characteristic arrangement of the collagen fibers parallel to the surface was not found. Therefore, this region can be assigned to the TZ instead of the SZ. Based on these results, it can be assumed that due to the late stage of OA, the SZ of the studied samples is mostly degraded.

Next, SEM was used to investigate the structural differences between non-infiltrated and infiltrated OA-cartilage explants (Figure 3D–F). SEM imaging was performed for three regions of the OA samples, as described above, and the same regions were compared (Figure 3G). In comparison to non-infiltrated OA-cartilage samples (controls), infiltrated OA-cartilage samples reveal characteristic deposits, which appear as part of a tissue–hydrogel composite, confirming successful tissue infiltration. These deposits were observed in all zones of MPC60-infiltrated OA-cartilage samples.

Representative, successful deposition of MPC60 hydrogel was observed in all investigated regions of the OA-cartilage plug (Figure 3D–F), whereas no such structures were observed in the controls (Figure 3A–C). The deposition of the MPC60 hydrogel in the center of the cartilage sample (TZ; Figure 3E) demonstrates exemplary complete infiltration for the zone with the longest infiltration pathway to the middle of the sample cross-section. Based on these results, we suggest that the infiltrated MPC60 monomer solution penetrates the whole tissue within 24 h. Thereby, an interpenetrating cartilage–hydrogel network is formed by the subsequent polymerization.

**Figure 4:**
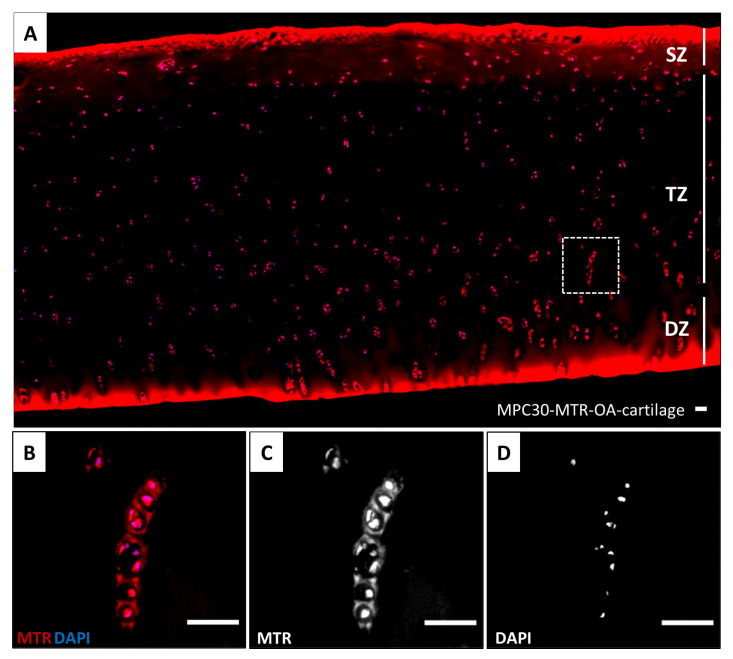
Fluorescence-based staining of human articular cartilage sections. (**A**) Human OA-cartilage explant, containing SZ, TZ and DZ, was entirely infiltrated with MPC30 monomer solution including the fluorescent dye MTR (red). Magnification 10×. (**B**) Chondrocytes and lacunae from the TZ (dashed box, **A**) infiltrated with MPC30 monomer solution (MTR, red) partially co-localized with cell nuclei which were stained with DAPI (blue). (**C**) Deposition of the hydrogel (red component) around the (**D**) cell nuclei (blue component). Magnification 40×. Scale bar 50 µm. n = 6. Magnification 4×. Scale bar 50 µm.

Additionally, fluorescence-based staining was performed to visualize the hydrogel-containing OA explants, exemplarily shown for OA-cartilage tissue containing MPC30 (Figure 4). A strong cell- and tissue-associated fluorescence signal (red) was detected, indicating successful and entire deposition of the hydrogel within the infiltrated cartilage structures from the SZ to the DZ (Figure 4A). Intense hydrogel deposits could be detected in the peripheries of the remaining SZ and the DZ (red, Figure 4A).

Hydrogels were mainly deposited in the cytosol of the chondrocytes, partially co-localized with the cell nuclei (Figure 4B,D) and pericellularly located in the chondrocyte lacunae (Figure 4B,C). No unspecific staining or autofluorescence of the OA-cartilage tissue and chondrocytes was detected when infiltrating OA cartilage with the monomer solutions without adding the fluorescent dye MTR (hydrogel control, Figure S1B in Supplementary Materials) or in OA-cartilage explants without hydrogel (OA-cartilage control, Figure S1B in Supplementary Materials). We did not observe any differences in hydrogel deposition between MPC60-, MPC30- and SMBA60-containing OA-cartilage tissues obtained from six patients.

In summary, these data from FTIR, SEM and fluorescence microscopy suggest successful hydrogel synthesis and a successful process of infiltration of the different zwitterionic monomer solutions (MPC30, MPC60, SBMA60) into human OA-cartilage explants within 24 h ex vivo.

### 3.2. Increased Toxicity of Monomer Solutions for Chondrocytes in 24 h Infiltrated OA-Cartilage Explants Compared to Embedded Isolated Chondrocytes

**Figure 5:**
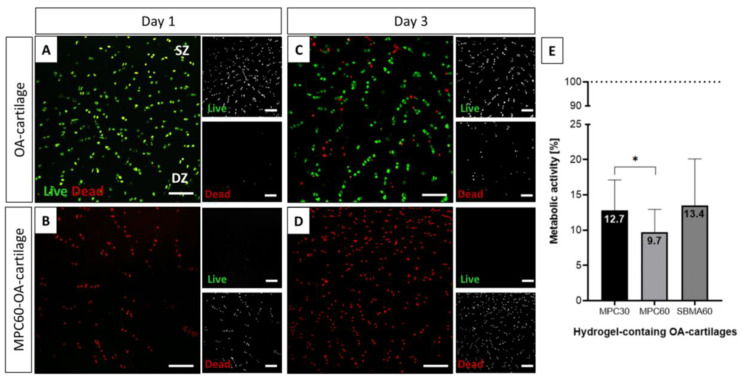
LIVE/DEAD Viability/Cytotoxicity assay of human OA-cartilage explants. Living cells of cross-sectional slices of human OA-cartilage tissue are stained with Ca-AM (green), and dead cells are stained with EthD-1 (red). (**A**) Chondrocytes of an untreated OA-cartilage explant (control) consist mostly of living cells after one day in culture, whereas (**B**) chondrocytes in OA-cartilage explants infiltrated with MPC60 monomer solutions for 24 h were mostly dead after one day in culture. (**C**) Chondrocytes of an untreated OA-cartilage explant (control) consist of living and dead cells, whereas (**D**) chondrocytes in OA-cartilage explants infiltrated with MPC60 hydrogel for 24 h were mostly dead after three days in culture. Human OA cartilage is classified into superficial zone (SZ) and deep zone (DZ); n = 5. Scale bar 200 µm. Magnification 20×. (**E**) Metabolic activity (days 1 and 3 combined) of chondrocytes in hydrogel-containing OA cartilage was evaluated in comparison to untreated OA cartilage (control, dotted line) using a CTB assay. Metabolic activity of chondrocytes was significantly decreased in infiltrated OA-cartilage explants (MPC30 = 12.7%, MPC60 = 9.7%, SBMA = 13.4%) compared to controls (100%). n = 5; * *p* < 0.05.

Potential cytotoxicity of infiltrated and polymerized MPC30, MPC60 and SBMA60 monomer solutions in OA-cartilage explants was analyzed based on LIVE/DEAD cell staining (exemplarily shown for MPC60, Figure 5A–D), and metabolic activity of chondrocytes in infiltrated OA-cartilage explants was measured using CTB assays (Figure 5E).

**Figure 6:**
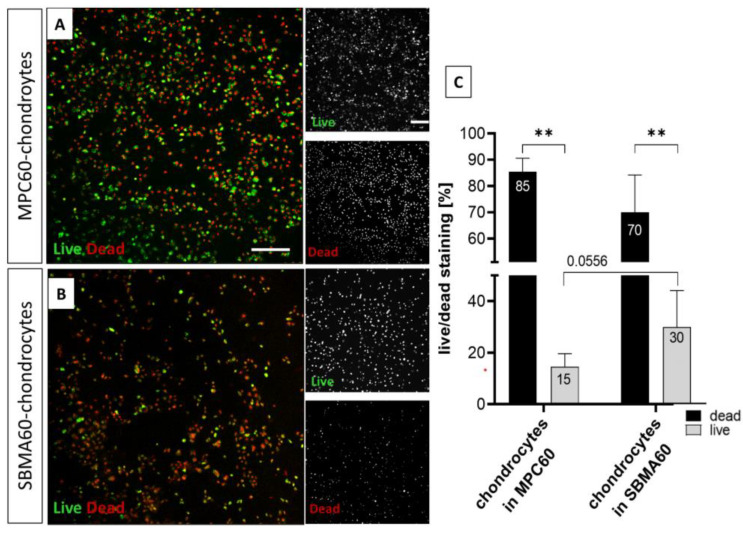
LIVE/DEAD Viability/Cytotoxicity assay of hydrogel-encapsulated isolated chondrocytes. Living cells of embedded chondrocytes were stained with Ca-AM (green), and dead cells were stained with EthD-1 (red). (**A**) Chondrocytes embedded in MPC60 hydrogel were partly living but mostly dead after one day in culture. (**B**) Chondrocytes embedded in SBMA60 hydrogel were partly living but mostly dead after one day in culture; n = 5. Scale bar 200 µm. Magnification 10×. (**C**) Ratio of living versus dead cells of all embedded chondrocytes (days 1–7 combined) in MPC60 and SBMA60 hydrogel showed significantly more dead cells (in MPC60 = 85%, in SBMA = 70%) than living cells (in MPC60 = 15%, in SBMA60 = 30%). In SBMA60 hydrogel, embedded chondrocytes showed a significantly (*p* = 0.0556) increased viability compared to embedded chondrocytes in MPC60 hydrogels; n = 5; ** *p* < 0.01; *p* = 0.0556.

The chondrocytes in untreated OA-cartilage explants (controls) were mostly viable after 1–3 days in culture (green, Figure 5A,C), although the number of dead cells was increased on day 3 (Figure 6C) compared to day 1 (Figure 6A). In contrast, chondrocytes of MPC60-containing OA explants were mostly dead 1 and 3 days in culture (Figure 5B,D). In addition, the metabolic activity of chondrocytes in hydrogel-containing cartilage explants was evaluated compared to the metabolic activity of chondrocytes in controls. MPC30 (12.7%), MPC60 (9.7%) and SBMA60 (13.4%) monomer solution infiltration and polymerization decreased metabolic activity of chondrocytes in OA-cartilage explants compared to non-hydrogel-containing OA samples (Figure 5E). We did not observe any differences in chondrocyte viability between MPC60-, MPC30- and SMBA60-containing OA-cartilage tissue from five patients (Figure S2 in Supplementary Materials). These results indicated cytotoxic effects of the infiltrated monomer solutions during 24 h prior to polymerization.

**Figure 7:**
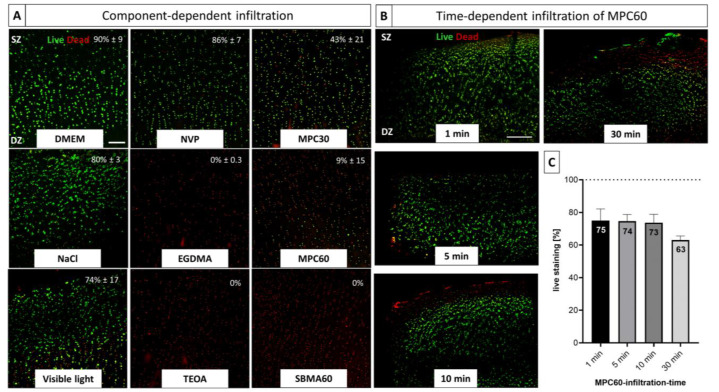
LIVE/DEAD Viability/Cytotoxicity assay of human OA-cartilage explants. Living cells in cross-sectional slices of human OA-cartilage tissue are stained with Ca-AM (green), and dead cells are stained with EthD-1 (red). (**A**) Chondrocytes of OA-cartilage explants were infiltrated with individual monomer solution components (DMEM, NVP, MPC30, NaCl, EGDMA, MPC60, TEOA, SBMA60) for 24 h (no light exposure) and subjected to irradiation with VL for 10 min. OA-cartilage tissues infiltrated with DMEM, NVP, NaCl and VL contained mostly living cells, whereas chondrocytes in OA-cartilage explants infiltrated with EGDMA, TEOA, MPC30, MPC60 and SBMA60 were mostly dead. Percent cell viability is given in white text with mean ± standard deviation; n = 3. Magnification 10×. Scale bar 200 µm. (**B**) Chondrocytes of OA-cartilage explants infiltrated with MPC60 monomer solution for different time periods (1, 5, 10 and 30 min) were mostly alive. With increased infiltration time, progressive cell death proceeding from the marginal areas of the OA explant is shown; n = 3. Magnification 4×. Scale bar 500 µm. (**C**) Living cells of chondrocytes in OA-cartilage explants containing MPC60 hydrogel after infiltration (1, 5, 10 and 30 min) were decreased with increased infiltration time (1 min = 75%, 5 min = 74%, 10 min = 73%, 30 min = 63%) compared to the total number of OA chondrocytes (100%, dotted line); n = 3. No significance.

In parallel to hydrogel-containing OA-cartilage explants, cell viability analysis using LIVE/DEAD cell staining assay was also performed for isolated primary OA chondrocytes embedded in MPC60 and SBMA60 hydrogels (Figure 6). The viability of the hydrogel-encapsulated chondrocytes was analyzed after one, three and seven days (Figure 6, Figure S4 in Supplementary Materials). Live and dead cells were observed in MPC60 and in SBMA60 hydrogels, representatively shown for day 1 (Figure 6A,B). However, the number of dead cells was significantly increased when chondrocytes were embedded in MPC60 and SBMA60 hydrogels compared to the number of living cells (Figure 6C). The comparison of MPC60- and SBMA60-embedded chondrocytes revealed that the viability of cells in SBMA60 hydrogels (30%) is significantly (*p* = 0.0556) higher than of the chondrocytes encapsulated in MPC60 hydrogels (15%) (Figure 7C). A viability assay of encapsulated chondrocytes in MPC30 hydrogels was not possible due to the low viscosity of the MPC30 hydrogels.

### 3.3. Time-Dependent and Component-Dependent Increase in Monomer Solution Cytotoxicity in Human OA-Cartilage Explants

Due to the high number of dead chondrocytes in entire infiltrated and polymerized cartilage–hydrogel composites, toxicity was also studied as a function of individual components of the hydrogel synthesis and of different infiltration times (1, 5, 10 and 30 min).

Analysis of the individual hydrogel components (Figure 7A) revealed that the cytotoxic effects can be attributed to the monomer solution components ethyleneglycol dimethacrylate (EGDMA), triethanolamine (TEOA) and the unpolymerized MPC30, MPC60 and SBMA60 monomers. However, a lower cytotoxicity was determined for the MPC30 solution (43 ± 21% viability) compared to MPC60 (9 ± 15% viability) and SBMA60 hydrogels (0% viability). Incubation in DMEM, PBS and N-vinylpyrrolidone (NVP) showed no significant influence on the cell viability, whereas irradiation with visible light had a slight cytotoxic effect on the marginal zones of the cartilage sample, resulting in a viability of 74% ± 17 (Figure 7A). Due to the autofluorescence of inactivated Eosin Y, the effects on chondrocyte viability could not be analyzed (Figure S3 in Supplementary Materials).

Since the single-component analysis for the different monomers, EGDMA and TEOA tended to exhibit cytotoxic effects within 24 h in human OA explants, the effects of reduced infiltration time points (1, 5, 10 and 30 min) were exemplarily investigated for MPC60 monomer solution (Figure 7B,C). Reducing the time of infiltration (1, 5, 10 and 30 min) resulted in increased cell viability but reduced infiltration depth compared to the samples infiltrated for 24 h (Figure 7B). With increasing infiltration time, decreased chondrocyte viability was observed (Figure 7C).

In summary, the monomer solutions have a component- and time-dependent effect on the viability of chondrocytes in OA-cartilage explants. The monomer solutions contain toxic components, the toxicity of which affects the whole tissue within 24 h. With a shortened infiltration time, it can be assumed that the tissue was infiltrated only in the marginal areas, which corresponds to the area of dead chondrocytes, although a vital tissue core was also preserved.

## 4. Discussion

Untreated cartilage lesions can lead to serious joint disorders such as osteoarthritis (OA). To date, there is no therapy available that is suitable as a treatment option in the early OA stages. Therefore, the establishment of new treatment approaches is of crucial importance. In this study, we developed a novel photo-crosslinking, zwitterionic sulfobetaine-methacrylate (SBMA)-based hydrogel and compared it with already studied 2-methacryloyloxyethyl-phosphorylcholine (MPC)-based hydrogels in terms of ex vivo infiltration into human OA-cartilage samples and their ability to encapsulate human isolated chondrocytes. The results indicated successful infiltration of zwitterionic MPC and SBMA monomer solutions into human OA-cartilage explants, and formation of a natural–synthetic network between OA-cartilage ECM and hydrogel through polymerization with visible light (VL). The evidence of complete deposition of the hydrogels within the OA-cartilage ECM supports the hypothesis that this proteoglycan-replacement hybrid system might be further optimized as a treatment option for cartilage defects to prevent or delay OA development.

### 4.1. Evidence of Polymerization and Infiltration

Crucial for the experimental design in this study was the proof of successful synthesis of MPC and SBMA hydrogels by polymerization of the monomer solutions with VL. The successful polymerization was indicated by the characteristic color change, which was also observed by Noshadi et al. [22]. Liquid monomer solutions are polymerized into hydrogels in the presence of photo-initiators mainly by using ultraviolet (UV) light [58]. Compared with the hazardous effects of UV radiation, such as damage to DNA and surrounding tissue, including accelerated aging, minimal damage is caused when using VL [58]. Therefore, VL is increasingly replacing UV light in in vitro and in vivo studies [59,60,61]. Cooper et al. [47] showed successful polymerization with VL for MPC monomer solutions, and Bahney et al. [62] reported the polymerization of hydrogels using VL with a photo-initiating system similar to that used in the present study, consisting of Eosin Y, TEOA and NVP.

In this study, the successful polymerization of the MPC monomer solutions and a novel SBMA monomer solution and their deposition within OA-cartilage explants were demonstrated by FTIR in addition to optical confirmation, as in comparable studies [22,63,64,65,66,67]. FTIR spectra of monomers, hydrogels and OA-cartilage explants with and without hydrogels were analyzed. Since the spectra of the investigated substances (OA cartilage, MPC or SBMA hydrogel) were known, the resulting peaks could be assigned to the characteristic functional hydrogel groups. Thus, polymerization and molecular deposition of MPC and SBMA hydrogels within the OA-cartilage explants were confirmed, in concordance with other studies [47,68].

Results of SEM and fluorescence microscopy provide a detailed understanding of the microstructure of infiltrated OA-cartilage explants. In the present study, the deposition of zwitterionic hydrogels within human OA-cartilage structures using SEM is shown for the first time. The observed orientation of the collagen fibers in the TZ (non-oriented) and the DZ (vertically oriented) could be verified, in accordance with other studies [56,57]. The initial parallel orientation of the collagen fibers, typical for the SZ, could not be found, which might be due to increasing degradation of the SZ during OA progression, as described in former studies [43,69,70]. Changoor et al. [57] were able to determine altered proportions of cartilage zones in degraded articular cartilage, characterized by an increase in the TZ. Hua et al. [68] demonstrated an interaction between porcine cartilage explants and hydrogel by using SEM; however, the hydrogel was not infiltrated into the explants, but solely applied to the cartilage surface.

For fluorescence microscopy. the MPC and SBMA monomer solutions were mixed with the fluorescent dye MTR, which allowed a detailed localization of the deposited hydrogels within the OA-cartilage zones, as already reported by Cooper et al. [47]. In the present study, complete hydrogel deposition within the entire OA explant could be detected, especially in and around chondrocytes and chondrons. The localization of the hydrogels near chondrocytes was also observed by Kowalski et al. [71]. Their hyaluronic acid-based hydrogel also accumulated in the region around the chondrocytes. It was reported that the increased deposition of the methacrylated hyaluronic acid gel correlates with the presence of type VI collagen within the ECM, which is an indicator for the pericellular matrix (PCM) of the chondrocytes [71,72]. This suggests that the MPC30, MPC60 and SBMA60 hydrogels of the present study preferentially accumulated at the PCM; however, this was not further investigated.

### 4.2. Biocompatibility

The used polymerized hydrogels appeared biocompatible, unlike the corresponding non-polymerized monomer solutions. Geever et al. [73] referred to increased cell toxicity due to monomer residues within polymerized hydrogels, but the cytotoxicity of zwitterionic monomer solutions is also in contrast to other studies. Bai et al. [74] and Dong et al. [35] showed a cell viability greater than 90% for encapsulated cells. In the present study, we observed that the cytotoxic effects of MPC and SBMA monomer solutions are due to one or more of the following non-polymerized components: TEOA, EGDMA, MPC60 and SBMA60. However, this is partly inconsistent with the results of other studies. Noshadi et al. [22] reported high cell viability for cardiomyocytes by using 1.5% TEOA, whereas Bahney et al. [62] confirmed a cytotoxic effect of TEOA for human mesenchymal stem cells. They recommended using a concentration of 0.1% instead of 1.5% TEOA to avoid toxic effects despite successful polymerization. The cytotoxicity of non-polymerized EGDMA was also determined by Bielecka et al. [75] for human gingival fibroblasts using an incubation time of 24 h. In contrast, high cell viability of isolated murine and human chondrocytes could be detected when EGDMA was pre-polymerized [76,77]. A study by Chien et al. [78] demonstrated that SBMA and MPC monomers exhibited low cytotoxic effects for a murine fibroblast cell line compared to other methacrylated monomers.

The predominance of encapsulated isolated dead chondrocytes in contrast to cartilage explants might be due to visible light irradiation. The isolated chondrocytes are no longer surrounded by their protecting ECM and are directly exposed to radiation which might be toxic then even if it is visible light. Several studies demonstrated the cytotoxic effects of blue light irradiation on isolated cells [79,80]. In contrast, the results of control OA-cartilage explants showed only a slight toxic impact on chondrocyte viability after irradiation with visible light for 10 min. Lim et al. [81] could also not find a negative influence of visible light irradiation on chondrocytes located within cartilage biopsies.

The hypothesis based on our results is that the isolated encapsulated chondrocytes exhibit increased cell viability compared to the chondrocytes located within the infiltrated OA-cartilage explants, which could be due to the reduced contact time with non-polymerized solutions. The infiltration and polymerization of hydrogels into human OA-cartilage explants within 24 h was successful, but the interaction of the unpolymerized monomer solutions and the OA chondrocytes within the cartilage tissue resulted in cytotoxicity. In comparison to cartilage explants, we observed an increased viability for the hydrogel-encapsulated OA chondrocytes. Polymerization of the embedded chondrocytes in the MPC and SBMA monomer solutions was performed immediately after mixing cells and monomer solutions, according to the literature [82]. Another study reported an incubation time of 10 min for cells and monomer solutions which achieved high cell viability; however, a different monomer solution was used [35]. A crucial contributing factor in this work was the incubation time (24 h) of the OA-cartilage explants with the monomer solutions. The focus of this work was a complete penetration of MPC and SBMA monomer solutions into human OA-cartilage explants. Due to the high number of dead cells after 24 h infiltration, some experiments were performed using reduced infiltration times (1, 5, 10 and 30 min). A higher chondrocyte viability in the innermost cartilage core was reached and can be considered as sealing or resurfacing of the degenerated OA-cartilage surface, as we assume that a reduced infiltration time does not lead to complete infiltration of the cartilage explant. Comparable studies used similar penetration time-points and detected also only a partial infiltration of the cartilage structures at the surface [71,83,84].

### 4.3. Translational Aspects

Regarding the reconstitution of destroyed OA-cartilage layers such as the superficial zone, or the encapsulation of chondrocytes within zwitterionic hydrogels, bioprinting is another promising treatment option for cartilage defects and early OA prevention. Through bioprinting, tissue structures can be generated in a defined manner. The composition of these so-called “bionics” is mostly based on natural or synthetic polymers, such as hydrogels, and can be dotted with different cells or bioactive molecules [85]. Cell-supported cartilaginous structures can be formed to support tissue regeneration by providing biomechanical stability. With regard to in situ treatments, a so-called “Biopen” has already been developed, which allows preparing multiple layers of different biomaterials, without or with cells (e.g., stem cells), to be inserted directly into the defect site and to be polymerized by the integrated light source [86,87]. The Biopen was already used by Onofrillo et al. [88] to generate human hyaline-like cartilage tissue by creating a scaffold with human adipose-derived mesenchymal stem cells, chondrogenic stimuli and gelatin-methacryloyl/hyaluronic acid methacryloyl hydrogels. Another regenerative strategy (bionic: gelatin-methacryloyl/hyaluronic acid methacryloyl and MSCs) was published by Di Bella et al. [87] who compared three methods for cartilage regeneration: 3D Biopen printed scaffolds, pre-constructed printed scaffolds and microfracturing in vivo. The defects treated with Biopen exhibited the highest amount of newly regenerated cartilage tissue compared to the other groups [86,87].

Hydrogel treatments of cartilage defects mainly focus on cartilage repair or replacement to replicate the biomechanical properties of the tissue, whereas chronic pain is also a crucial symptom of OA that needs to be addressed. Structure-based therapies with DMOADs, pain-based therapies with NSAIDs or opioids, and cell-based options with stem cells represent promising treatment options [8]. Could a combined therapy involving DMOADs, NSAIDs and/or stem cells encapsulated in monomer solutions and/or hydrogels to restore mechanical stability, repair and regenerate degenerated cartilage and eventually relieve pain be a potential new treatment option for degenerative joint diseases?

## 5. Concluding Remarks

In summary, we have shown that zwitterionic-based hydrogels (MPC and SBMA) are suitable for the infiltration of human OA-cartilage samples. The applied hydrogels could completely infiltrate the human cartilage explants and still be polymerized by visible light. The interpenetrating network of hydrogel and cartilage was shown by SEM and fluorescence microscopy, and the polymerization was confirmed by FTIR. Furthermore, the monomer solutions have shown a cytotoxic effect in viability tests during infiltration. This effect could be reduced by sealing the surface instead of completing the entire infiltration. Based on the results of the present study and the already-known advantages of zwitterionic polymers (anti-fouling, lubricant, mechanical properties), we conclude that the generated hydrogels have great potential in terms of sealing and resurfacing of degraded OA-cartilage explants. Further studies are needed to investigate the mechanical and swelling behavior, as well as lubricating, anti-fouling and long-term infiltration properties of the hydrogel–cartilage explants. The generated MPC and SBMA hydrogels might be also used as bionics, with or without embedded chondrocytes, in terms of OA-cartilage regeneration.

## Data Availability

The data supporting the reported findings of this study are available from the corresponding author upon reasonable request.

## Supplementary Materials

The following supporting information can be downloaded at: https://www.mdpi.com/article/10.3390/bioengineering10070767/s1, Figure S1: Fluorescence-based staining of human articular cartilage sections. Figure S2: LIVE/DEAD Viability/Cytotoxicity assay of human OA-cartilage explants. Figure S3: LIVE/DEAD Viability/Cytotoxicity assay of unpolymerized Eosin Y within human OA-cartilage explants. Figure S4: LIVE/DEAD Viability/Cytotoxicity assay of isolated encapsulated OA chondrocytes in hydrogels. Table S1. Listing of used OA-patient samples including sex, age, and associated experiment.
