# Supplementary material for: In Vitro Analysis of Human Cartilage Infiltrated by Hydrogels and Hydrogel-Encapsulated Chondrocytes
Source: Bioengineering (Basel). 2023 Jun 26;10(7):767. doi: 10.3390/bioengineering10070767 (PMC10376441; doi:10.3390/bioengineering10070767)
Supplement: Supplementary file 1 [file bioengineering-10-00767-s001.zip › bioengineering-2333972-supplementary.pdf]

Supplementary Materials

In vitro Analysis of Human Cartilage Infiltrated by Hydrogels and Hydrogel-Encapsulated Chondrocytes

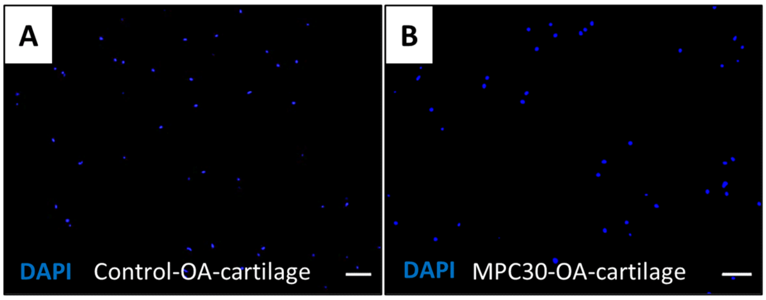

Figure S1. Fluorescence based staining of human articular cartilage sections. No red background staining was observed in human OA-cartilage sections **(A)** without both MTR and MPC30-hydrogel and **(B)** exemplary with MPC30-hydrogel but without MTR; n = 6. Magnification 4×. Scale bar 50 µm.

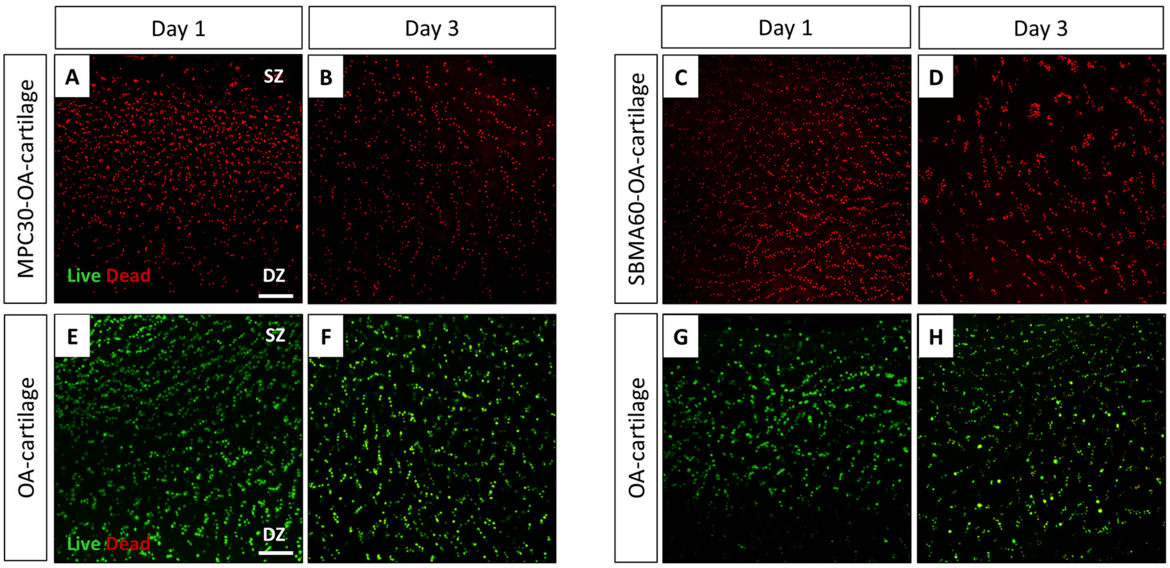

Figure S2. LIVE/DEAD® Viability/Cytotoxicity assay of human OA-cartilage explants. Living cells of cross-sectional slices of human OA-cartilage tissue are stained with Ca-AM (green) and dead cells are stained with EthD-1 (red). (**A,B**) Hydrogels containing isolated chondrocytes from OA-cartilage explants which were infiltrated with MPC30-monomer solutions for 24h and subsequently polymerized, showed mostly dead cells after one and three days in culture. (**C,D**) OA-cartilage explants which were infiltrated with SBMA60-monomer solutions for 24h and subsequently polymerized show mostly dead chondrocytes after one and three days in culture. (**E–H**) Chondrocytes of OA-cartilage without hydrogels consisted mostly of living cells after one and three days in culture. Human OA-cartilage is classified in SZ (superficial zone) and DZ (deep zone). n = 5; magnification 10×. Scale bar 200 µm.

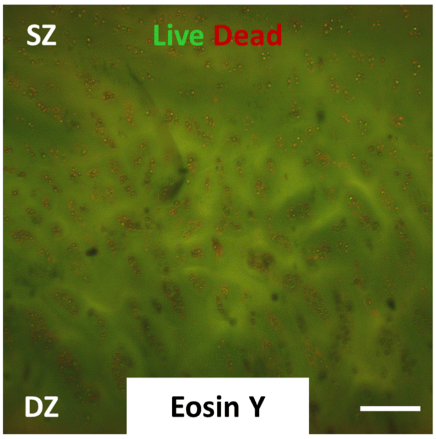

Figure S3. LIVE/DEAD® Viability/Cytotoxicity assay of unpolymerized Eosin Y within human OA-cartilage explants. Due to the autofluorescence of inactivated Eosin Y, living and dead cells cannot be identified. Human OA-cartilage is classified in SZ (superficial zone) and DZ (deep zone). n = 3; magnification 10×. Scale bar 200 µm.

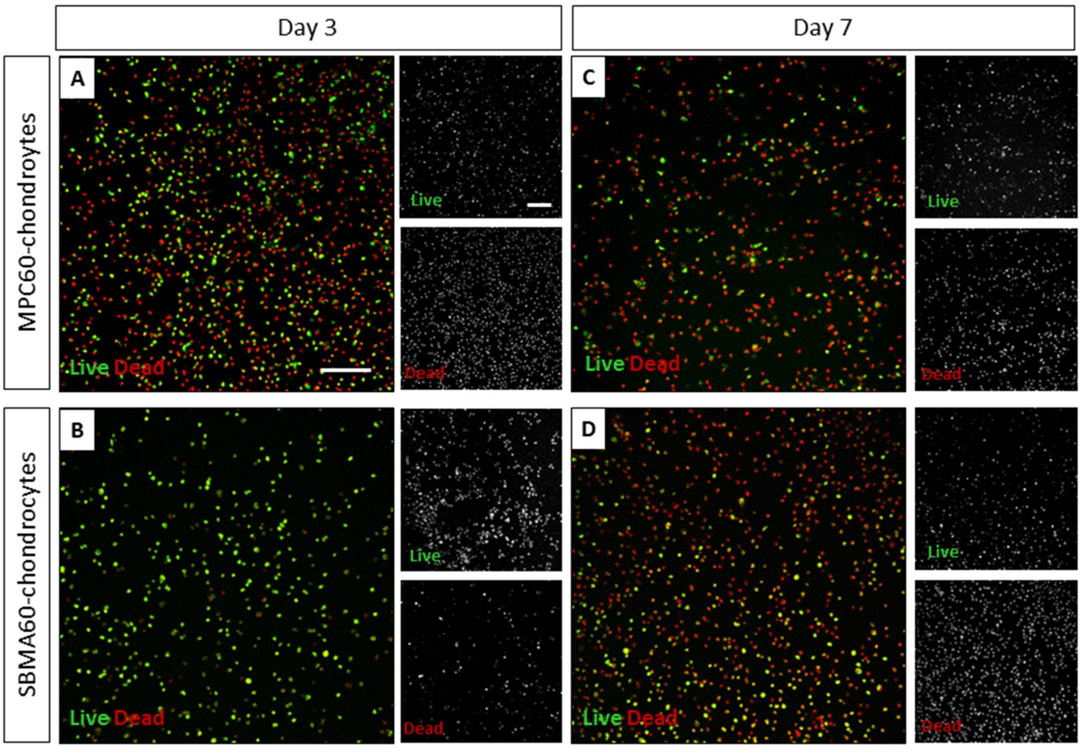

Figure S4. LIVE/DEAD® Viability/Cytotoxicity assay of isolated encapsulated OA-chondrocytes into hydrogels. Living chondrocytes are stained with Ca-AM (green) and dead cells are stained with EthD-1 (red). (**A,C**) MPC60-hydrogel contained scattered living but mostly dead cells after three and seven days in culture. (**B,D**) SBMA60-hydrogels showed also living and dead cells after three and seven days in culture. Insets on the right side show EthD-1 (live) and Ca-AM (dead) individually. Human OA-cartilage is classified in SZ (superficial zone) and DZ (deep zone). n = 5; magnification 10×. Scale bar 200 µm.

Table S1. Listing of used OA-patient samples including sex, age, and associated experiment.

OA-patient	sex	age	experiment
1	mal	58	FTIR_MPC60 (3.1)
2	male	71	FTIR_MPC60 (3.1)
3	male	74	FTIR_MPC60 (3.1)
4	female	79	FTIR_MPC60 (3.1)
5	male	78	FTIR_MPC60 (3.1)
6	male	50	FTIR_MPC60 (3.1)
7	female	79	FTIR_MPC30 (3.1)
8	male	50	FTIR_MPC30 (3.1)
9	male	74	FTIR_MPC30 (3.1)
10	male	78	FTIR_MPC30 (3.1)
11	male	71	FTIR_MPC30 (3.1)
12	female	73	FTIR_MPC30 (3.1)
13	male	71	FTIR_SBMA60 (3.1)
14	female	82	FTIR_SBMA60 (3.1)
15	male	73	FTIR_SBMA60 (3.1)
16	male	73	FTIR_SBMA60 (3.1)
17	male	78	FTIR_SBMA60 (3.1)
18	male	74	FTIR_SBMA60 (3.1)
19	male	62	SEM_MPC60 (3.1)
20	male	58	SEM_MPC60 (3.1)
21	female	82	SEM_MPC60 (3.1)
22	female	73	SEM_MPC60 (3.1)
23	female	67	SEM_MPC60 (3.1)
24	female	78	SEM_MPC60 (3.1)
25	male	40	Fluorescence microscopy_MPC60 (3.1)
26	male	62	Fluorescence microscopy_MPC60 (3.1)
27	female	63	Fluorescence microscopy_MPC60 (3.1)
28	male	58	Fluorescence microscopy_MPC60 (3.1)
29	female	62	Fluorescence microscopy_MPC60 (3.1)
30	female	88	Fluorescence microscopy_MPC60 (3.1)
31	male	80	Fluorescence microscopy_MPC30 (3.1)
32	male	84	Fluorescence microscopy_MPC30 (3.1)
33	female	88	Fluorescence microscopy_MPC30 (3.1)
34	male	50	Fluorescence microscopy_MPC30 (3.1)
35	male	78	Fluorescence microscopy_MPC30 (3.1)
36	female	79	Fluorescence microscopy_MPC30 (3.1)
37	female	56	Fluorescence microscopy_SBMA60 (3.1)
38	female	62	Fluorescence microscopy_SBMA60 (3.1)
39	male	50	Fluorescence microscopy_SBMA60 (3.1)
40	male	78	Fluorescence microscopy_SBMA60 (3.1)
41	female	79	Fluorescence microscopy_SBMA60 (3.1)
42	Female	62	Fluorescence microscopy_SBMA60 (3.1)

43	male	78	OA-cartilage_CLSM_livedead+CTB_MPC60 (3.2)
44	female	71	OA-cartilage_CLSM_livedead+CTB_MPC60 (3.2)
45	male	82	OA-cartilage_CLSM_livedead+CTB_MPC60 (3.2)
46	female	68	OA-cartilage_CLSM_livedead+CTB_MPC60 (3.2)
47	female	68	OA-cartilage_CLSM_livedead+CTB_MPC60 (3.2)
48	male	79	OA-cartilage_CLSM_livedead+CTB_MPC30 (3.2)
49	female	74	OA-cartilage_CLSM_livedead+CTB_MPC30 (3.2)
50	male	65	OA-cartilage_CLSM_livedead+CTB_MPC30 (3.2)
51	male	55	OA-cartilage_CLSM_livedead+CTB_MPC30 (3.2)
52	male	61	OA-cartilage_CLSM_livedead+CTB_MPC30 (3.2)
53	female	74	OA-cartilage_CLSM_livedead+CTB_SBMA60 (3.2)
54	female	71	OA-cartilage_CLSM_livedead+CTB_SBMA60 (3.2)
55	female	70	OA-cartilage_CLSM_livedead+CTB_SBMA60 (3.2)
56	male	74	OA-cartilage_CLSM_livedead+CTB_SBMA60 (3.2)
57	male	75	OA-cartilage_CLSM_livedead+CTB_SBMA60 (3.2)
58	female	49	Chondorcytes_CLSM_livedead+CTB_MPC60 (3.2)
59	female	47	Chondorcytes_CLSM_livedead+CTB_MPC60 (3.2)
60	female	52	Chondorcytes_CLSM_livedead+CTB_MPC60 (3.2)
61	male	52	Chondorcytes_CLSM_livedead+CTB_MPC60 (3.2)
62	male	58	Chondorcytes_CLSM_livedead+CTB_MPC60 (3.2)
63	female	49	Chondorcytes_CLSM_livedead+CTB_SBMA60 (3.2)
64	male	52	Chondorcytes_CLSM_livedead+CTB_SBMA60 (3.2)
65	female	83	Chondorcytes_CLSM_livedead+CTB_SBMA60 (3.2)
66	female	85	Chondorcytes_CLSM_livedead+CTB_SBMA60 (3.2)
67	female	73	Chondorcytes_CLSM_livedead+CTB_SBMA60 (3.2)
68	female	81	Component analyses CLSM_livedead (3.3)
69	male	70	Component analyses CLSM_livedead (3.3)
70	male	55	Component analyses CLSM_livedead (3.3)
71	female	72	time analyses CLSM_livedead (3.3)
72	female	71	time analyses CLSM_livedead (3.3)
73	male	73	time analyses CLSM_livedead (3.3)
